# Propensity score matched analysis of postoperative nausea and pain after one anastomosis gastric bypass (MGB/OAGB) versus sleeve gastrectomy (SG)

**DOI:** 10.1007/s13304-023-01536-1

**Published:** 2023-05-16

**Authors:** Antonio Vitiello, Carmine Iacovazzo, Giovanna Berardi, Maria Vargas, Annachiara Marra, Pasquale Buonanno, Nunzio Velotti, Mario Musella

**Affiliations:** 1 Advanced Biomedical Sciences Department, Naples “Federico II” University, AOU “Federico II”-Via S. Pansini 5, 80131 Naples, Italy; 2 Department of Neurological, Reproductive and Odontostomatological Sciences, Naples “Federico II” University, AOU “Federico II”-Via S. Pansini 5, 80131 Naples, Italy

**Keywords:** Anaesthesia, Nausea, Pain, Sleeve gastrectomy, One anastomosis gastric bypass

## Abstract

The aim of our study was to assess and compare postoperative nausea and pain after one anastomosis gastric bypass (OAGB) and sleeve gastrectomy (LSG). Patients undergoing OAGB and LSG at our institution between November 2018 and November 2021 have been prospectively asked to report postoperative nausea and pain on a numeric analogic scale. Medical records were retrospectively reviewed to collect scores of these symptoms at the 6th and 12th postoperative hour. One-way analysis of variance (ANOVA) was used to evaluate effect of type of surgery on postoperative nausea and pain scores. To adjust for baseline differences between cohorts, a propensity score algorithm was used to match LSG patients to MGB/OAGB patients in a 1:1 ratio with a 0.1 tolerance. A total number of 228 (119 SGs and 109 OAGBs) subjects were included in our study. Nausea after OAGB was significantly less severe than after LSG both at the 6th and 12th hour assessment; pain was less strong after OAGB at the 6th hour but not after 12 h. Fifty-three individuals had a rescue administration of metoclopramide after LSG and 34 after OAGB (44.5% vs 31.2%, *p* = 0.04); additional painkillers were required by 41 patients after LSG and 23 after OAGB (34.5% vs 21.1%, *p* = 0.04). Early postoperative nausea was significantly less severe after OAGB, while pain was comparable especially at the 12th hour.

## Introduction

The laparoscopic sleeve gastrectomy (LSG) is currently the most common bariatric procedure worldwide [1], while the one anastomosis gastric bypass (OAGB) represents the third intervention in Europe [2].

Although there is a rich body of literature on postoperative nausea and vomit (PONV) after LSG [3], very little is available on OAGB.

Assessment of these symptoms is particularly important since PONV occurs more frequently after bariatric surgery than after other abdominal surgical procedures [4–6].

Moreover, PONV is responsible for prolonged hospital stay and 30-day readmission [7, 8]

Among all weight loss interventions, LSG is undoubtedly the most emetogenic [9] one, probably due to the high intraluminal pressure in the sleeved stomach.

Another main issue in the first postoperative hours is pain. Forty percent of patients with morbid obesity complains with severe postoperative pain which can lead to pulmonary complications and increased risk of thromboembolism due to immobility [10, 11].

To reduce the rate of PONV and the severity of postoperative pain, a specific Early Recovery After Bariatric Surgery (ERABS) protocol has been designed [12, 13].

In our institution, ERABS guidelines are not strictly followed, indeed a nasogastric tube is routinely used and total intravenous anaesthesia (TIVA), instead of volatile anaesthetics, is not given to all patients. However, subjects with morbid obesity undergoing bariatric surgery in our hospital receive the same perioperative management.

The aim of this study is to retrospectively compare postoperative nausea and pain after OAGB and LSG.

## Methods

All subjects that have undergone primary OAGB and LSG between 1st November 2018 and 1st November 2021 at our university hospital were included in this study. Those individuals who had an early complication or a concomitant procedure (hiatal hernia repair, abdominal wall reconstruction, cholecystectomy), with previous history of abdominal surgery or transferred to the Intensive Care Unit (ICU) immediately after surgery were excluded.

Patients undergoing bariatric surgery at our institution are routinely asked to report their pain and nausea on a numeric scale (0 = *not at all* to 10 = *worst imaginable nausea/pain*).

Medical records were retrospectively reviewed to collect data on preoperative sex, age, body mass index (BMI), pain and nausea at the 6th and 12th postoperative hour and preoperative symptoms of gastro-esophageal reflux disease (GERD).gastro-esophageal reflux disease (GERD) was diagnosed according to the Lyon Consensus Conference [14] criteria in case of preoperative heartburn and regurgitation. Individuals with esophagitis > B according to the Los Angeles Classification [15] are submitted to Roux-en-Y Gastric Bypass (RYGB).

The choice of the procedure (OAGB or LSG) in our centre is based on patients’ BMI and obesity related diseases. Those individuals with higher BMI or metabolic complications such as Diabetes are more likely to undergo OAGB.

### Surgical technique

Surgical techniques for both procedures have been described in detail elsewhere [16, 17],but a brief description is reported below for completeness of the article. Patient was placed in the reverse Trendelenburg position.

For LSG, a five-trocar approach (3 × 12 mm, 2 × 5 mm) was used. The gastrectomy started 4–6 cm from the pylorus over a 38–40 French bougie. Staple line reinforcements or oversewing is not routinely used at our institution.

OAGB was routinely performed with a six-port laparoscopic technique. The gastric pouch was constructed by applying one horizontal 45-mm linear stapler at the lesser curvature, just below the left branch of the crow’s foot. Biliopancreatic limb length ranged from 180 to 220 cm depending on the preoperative BMI of patients. All the anastomoses were performed at least 13 cm distally to the GEJ.

### Anaesthesia and Postoperative care

After admission to the operating room two intravenous cannulas (16/18-gauge) were inserted; patients were monitored using a five-lead ECG, invasive arterial pressure monitoring, pulse oximeter, capnograph, end-tidal anaesthetic gas (ETAG) concentration monitoring, urine output and temperature. After proper assessment of the airway and anticipation of difficult airway, preoxygenation with 100% O_2_ on 8 L/min for 3 min via face mask in ramped position was started. Induction was performed with propofol 2 mg/Kg of lean body weight, fentanyl 2–5 mcg/kg of lean body weight, rocuronium 0.6 mg/kg of ideal body weight followed by intubation. Muscle relaxation was monitored through train-of-four (TOF). Ventilation was performed with tidal volumes of 6–8 mL/Kg to avoid barotraumas and respiratory rates 12–14 breaths/min to maintain normocapnia and Positive End Expiratory Pressure (PEEP) of 5–10 cmH_2_O. Anaesthesia was maintained with desflurane with a MAC between 0.6 and 1, remifentanil infusion of 0.05–0.25 mcg/kg/min of lean body weight. No abdominal wall block or intraoperative injection of local anaesthetics was performed. Postoperative analgesia was provided with a continuous intravenous administration of ketorolac 90 mg, ondansetron 8 mg and oxycodone 10 mg through an elastomeric infusion pump. All patients assumed Paracetamol 1 g iv every 6 h for 24 as a rescue drug. Intravenous metoclopramide 10 mg was administered in case of severe nausea.

A nasogastric tube was placed and left for the first 24 h after both interventions; liquid diet was started on postoperative day (POD) 3. Subjects were mobilized on POD1.

### Statistical analysis

Data are expressed as mean ± SD for continuous variables and as proportion or percentage in case of categorical ones. Continuous and categorical variables were compared using the chi-square and t-test, respectively. One-way analysis of variance (ANOVA) was used to evaluate effect of type of surgery on postoperative nausea and pain scores. To adjust for baseline differences between cohorts, a propensity score algorithm was used to match LSG patients to MGB/OAGB patients in a 1:1 ratio with a 0.1 tolerance. Propensity score matching (PSM) is a well-validated statistical technique that creates comparable groups and allows for accurate assessment of treatment effects. Patients were matched for preoperative age, BMI and GERD.

Significant p value was set below 0.05. Data analyses were performed using Statistical Package for Social Science for Windows, version 28 (SPSS Inc., Chicago, IL, USA).

## Results

A total number of 136 primary OAGBs and 178 primary SGs have been performed at our institution in the study period. On the base of exclusion/inclusion criteria, 51 cases (early complication or concomitant procedure) were not eligible for this study, while data on postoperative nausea and pain were not available for 35 patients. Subsequently, 228 (119 SGs and 109 OAGBs) subjects were included in our retrospective comparison. After PSM, two matched groups of 73 subjects each were generated.

Patients undergoing the two procedures had comparable age, rate of GERD and female/male ratio at baseline, but BMI was significantly higher in the OAGB group; all preoperative variables resulted comparable at baseline between the matched groups (Table 1).

**Table 1:** Baseline demographics of the two groups with and without propensity score matching

	Unmatched analysis		*P* value	Matched analysis		*P* value
	OAGB (*N* = 109)	LSG (*N* = 119)		OAGB (*N* = 73)	LSG (*N* = 73)	
Age (Years)	39.8 ± 9.9	38.4 ± 10.2	0.3	39.5 ± 10.1	39.4 ± 9.9	*0.9*
BMI (Kg/m2)	45.9 ± 6.5	43.5 ± 5.2	0.002	44 ± 6	44.3 ± 5.4	*0.7*
GERD	48 (44%)	43 (36%)	0.2	31 (42%)	28 (38%)	*0.4*
SEX (F)	67 (61%)	81 (68%)	0.3	26 (34%)	20 (27%)	*0.3*

*SG* sleeve gastrectomy; *OAGB* one anastomosis gastric bypass; *BMI* body mass index

Overall values of nausea at the 6th and 12th hour were 5.8 ± 1.2 and 3.7 ± 1.3 respectively, while total scores for pain were 5.9 ± 1.1 and 3 ± 1.

Nausea after OAGB was significantly less severe than after LSG both at the 6th- and 12th- hour assessment before and after PSM. On the contrary pain was significantly less strong after OAGB at the 6^th^ hour but not after 12 h before PSM, while no significant difference for pain was found between the matched groups (Table 2).

**Table 2:** Nausea and pain scores at 6 and 12 h after OAGB and LSG with and without propensity score matching

	Unmatched analysis					Matched analysis			
	Type of surgery	Mean	Standard deviation	*P* value		Type of surgery	Mean	Standard deviation	*P* value
NAUSEA_6	OAGB	5.2	1.3	< *0.01*	NAUSEA_6	OAGB	5.4	1.3	< *0.01*
	LSG	6.3	0.7			LSG	6.2	0.7	
NAUSEA_12	OAGB	2.9	1.1	< 0.01	NAUSEA_12	OAGB	3	1.1	< 0.01
	LSG	4.4	1			LSG	4.3	0.9	
PAIN_6	OAGB	5.6	1.4	< 0.01	PAIN_6	OAGB	5.8	1.4	0.09
	LSG	6.1	0.6			LSG	6.1	0.6	
PAIN_12	OAGB	2.9	1.2	0.05	PAIN_12	OAGB	3.1	1.2	0.7
	LSG	3.2	0.8			LSG	3.2	0.8	

## Discussion

### Nausea

Postoperative nausea and vomiting negatively affects early hours after LSG with a reported incidence up to 90% [18]. Occurrence of this symptoms is lower after RYGB, especially when ERABS protocol is used [19]. Even if PONV is usually self-limiting, when this condition persists it has a negative impact on patient satisfaction, hospital stay and risk of readmission [20].

For these reasons, different combinations of perioperative drugs have been suggested to reduce nausea and vomiting after bariatric surgery [21, 22].

In our experience all patients undergoing LSG and OAGB experienced a variable degree of early nausea. Vomiting was not assessed in our retrospective analysis due to the routine use of a nasogastric tube, which is not recommended by the ERABS guidelines [23] and it could have biased the outcomes. Therefore, in our study only nausea was considered as a symptom of delayed functional recovery of the stomach.

Female sex and use of volatile anaesthetics are usually associated with PONV [24]; in our cohort of patient there was no difference in female/male ratio in the two groups and only desflurane, whose efficacy in bariatric patients is comparable to TIVA [25], was used as a volatile anaesthetic.

As reported in the current literature, our data have further confirmed that nausea tends to significantly reduce in the first 12 postoperative hours. Moreover, our comparison demonstrated that regardless preoperative demographics, this symptom was significantly less severe after OAGB. Indeed, after PSM, nausea was significantly more severe after LSG both at the 6th- and 12th-hour assessment. This finding also correlates with the lower rate of rescue medicines used in the postoperative period after OAGB, which could subsequently be considered as a better option for patients with preoperative GERD.

### Pain

Uncontrolled postoperative pain has a detrimental effect on respiratory function, mobility, thromboembolic complications, nausea and vomiting [26]. Unfortunately, management of this symptom is a demanding task in patients with morbid obesity [27].

Moreover, excessive use of painkillers during the early hours after surgery increases the risk of chronic post-surgical pain and opioid dependence [28].

Several strategies, such as transversus abdominis plane (TAP) block [29] or multimodal intraoperative administration of different drugs [11, 30], have been proposed to optimize pain management after bariatric surgery.

In our institution a standardized continuous intravenous injection of painkillers is preferred, and opioids are forbidden as rescue medicines. Before PSM, intensity of this symptom was lower after OAGB at the 6th, but no significant difference was found after matching. Even if the resection and removal of 80% of the stomach induced worse early distress, this was adequately controlled in the first 12 h by our protocol. Indeed, this greater postoperative discomfort after LSG was also demonstrated by the higher rate of additional painkillers administered postoperatively.

### Strength and limitations

ERABS guidelines are not strictly followed in our department, therefore a nasogastric tube is routinely placed and oxycodone (elastomeric pump) was administered postoperatively to all patients. Nausea and pain assessments relied totally on a self-reported numeric scale and no validated questionnaire was used. Being this study retrospective, possible confounders and heterogeneity related to preoperative selection bias, which we tried to reduce using PSM. However, this is the first report of these symptoms after OAGB in a large cohort of patients undergoing the same perioperative management.

## Conclusion

Early postoperative nausea was significantly more severe after LSG rather than after OAGB with a greater percentage of patients requiring rescue medicines.

Pain was more intense after LSG at the 6th hour with higher rate of additional painkillers administered in this group. Postoperative discomfort was comparable after PSM.
